# Identification of Importin 8 (IPO8) as the most accurate reference gene for the clinicopathological analysis of lung specimens

**DOI:** 10.1186/1471-2199-9-103

**Published:** 2008-11-17

**Authors:** Paul A Nguewa, Jackeline Agorreta, David Blanco, Maria Dolores Lozano, Javier Gomez-Roman, Blas A Sanchez, Iñaki Valles, Maria J Pajares, Ruben Pio, Maria Jose Rodriguez, Luis M Montuenga, Alfonso Calvo

**Affiliations:** 1 Division of Oncology, Center for Applied Medical Research (CIMA), University of Navarra, Avda, Pio XII, 55, 31008 Pamplona, Spain; 2 Department of Pathology, University Hospital of Navarra, Avda, Pio XII, 36, 31008 Pamplona, Spain; 3 Department of Anatomical Pathology, Marqués de Valdecilla University Hospital, Medical Faculty, University of Cantabria, Santander, Spain; 4 Research Department, Ingenasa, Madrid, Spain

## Abstract

**Background:**

The accurate normalization of differentially expressed genes in lung cancer is essential for the identification of novel therapeutic targets and biomarkers by real time RT-PCR and microarrays. Although classical "housekeeping" genes, such as GAPDH, HPRT1, and beta-actin have been widely used in the past, their accuracy as reference genes for lung tissues has not been proven.

**Results:**

We have conducted a thorough analysis of a panel of 16 candidate reference genes for lung specimens and lung cell lines. Gene expression was measured by quantitative real time RT-PCR and expression stability was analyzed with the softwares *GeNorm *and *NormFinder*, mean of |ΔCt| (= |Ct Normal-Ct tumor|) ± SEM, and correlation coefficients among genes. Systematic comparison between candidates led us to the identification of a subset of suitable reference genes for clinical samples: IPO8, ACTB, POLR2A, 18S, and PPIA. Further analysis showed that IPO8 had a very low mean of |ΔCt| (0.70 ± 0.09), with no statistically significant differences between normal and malignant samples and with excellent expression stability.

**Conclusion:**

Our data show that IPO8 is the most accurate reference gene for clinical lung specimens. In addition, we demonstrate that the commonly used genes GAPDH and HPRT1 are inappropriate to normalize data derived from lung biopsies, although they are suitable as reference genes for lung cell lines. We thus propose IPO8 as a novel reference gene for lung cancer samples.

## Background

Lung cancer is one of the most fatal types of cancer in the world. The overall 5-yr survival rate remains at 15%, as most patients present with advanced disease [1]. The prognosis for the patients is highly correlated to the stage of disease at the time of diagnosis. Lung cancer is usually diagnosed in an advanced stage, which is frequently too late for surgical intervention, and therefore, it usually becomes incurable.

During the past few years, the application of microarray technology has revolutionized cancer genomics, making possible the simultaneous evaluation of the expression of thousands of genes. Newly discovered gene signatures in lung [2] and breast cancer [3] may predict disease outcome and contribute to the design of novel therapeutic targets. The use of gene expression profiles in routine clinical practice is highly dependent on precise identification and robust validation of these gene signatures, which relies upon a high-throughput RT-PCR-based technology is available.

Quantitative real time PCR (qRT-PCR) is one of the most powerful quantification methods for gene expression analysis. This technology has been applied to identify molecular tumor biomarkers [4], splice variants of target genes [5], and microRNAs [6], and to quantify circulating DNA [7], with the final goal of improving diagnosis and predicting clinical outcome [8]. In these studies, target gene expression is usually quantified in relation to a stably expressed reference gene, simultaneously determined in the sample [9]. Although it is assumed that these reference genes are constitutively expressed in certain tissues and under certain circumstances, the literature shows that the expression levels of some of the "classic" endogenous control genes may in fact vary in different tissues, cell types, and disease stages [10]. It is then clear that if US Food and Drug Administration (FDA) or other Regulatory Agencies are to approve any diagnostic or prognostic test based on qRT-PCR, the proof of the stability of the proposed reference genes will be a major requirement. Therefore, the selection of suitable reference genes is a key prerequisite to control the variability of clinical samples.

Recent lung cancer molecular profiling studies have employed a group of widely used endogenous control genes, such as GAPDH [11], beta-actin (ACTB) [12], TATA-binding protein (TBP) [4], 18s-rRNA [13], HMBS [5] and phenylalanine hydroxylase [14], for RT-PCR. Such genes were selected in the past as reference genes for non- or semi-quantitative techniques and have been used for many years in most experiments to measure qualitative gene expression changes. These widely used reference genes were not selected for specific tissue types or organs and were mainly validated in cell lines. The advent of qRT-PCR allows for the accurate quantification of expression changes, albeit some studies have continued using these old reference genes without a re-evaluation of their suitability as endogenous control genes. The requirement for a specific validation of the currently used reference genes is compelling and the need for robust stable endogenous genes for lung cancer is urgent.

We studied a panel of sixteen genes (some of them frequently used as endogenous controls) and analyzed their suitability as reference genes in both lung cell lines and clinical lung samples. From those, we identified *Importin 8 *(IPO8) as the most suitable gene for normalizing clinical lung specimens.

## Methods

### Tumor tissues and cultured cells

**Figure 1:**
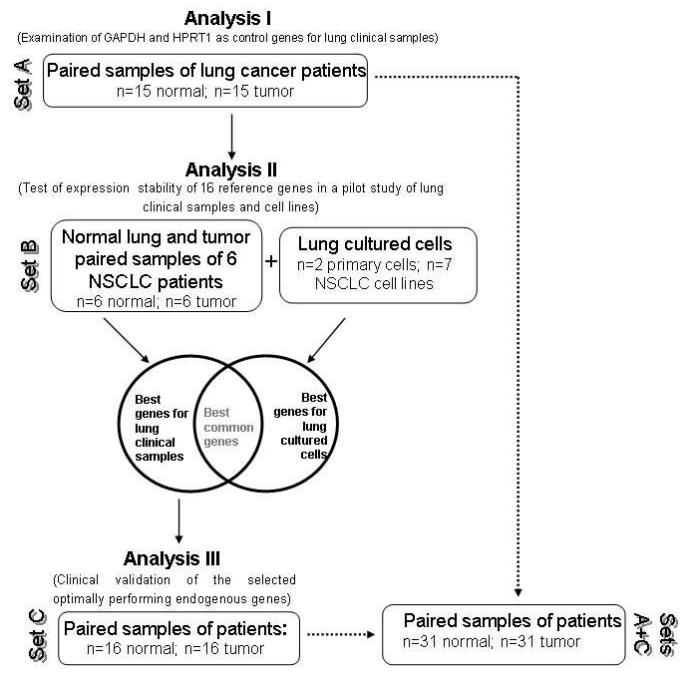
**Schematic diagram of the overall procedure for the identification of accurate reference genes**. General strategy to identify the most accurate reference genes for lung cancer mRNA quantification analysis in three different sets of samples.

Tumor samples were obtained from Non-Small Cell Lung Cancer (NSCLC) patients who underwent tumor resectional surgery at the University Hospital of Navarra (Pamplona, Spain) and at the Hospital Marqués de Valdecilla (Santander, Spain), under approved ethical protocols and informed consent from each patient (See Supplemental Table 1, Additional file 1). Surgically removed samples (the tumor and its corresponding matched normal tissue) were snap-frozen in liquid nitrogen. A 5 μm section was cut with a cryostat, and analyzed by histology. The general strategy of our analysis is illustrated in Figure 1. In a first study, consisting in the analysis of GAPDH and HPRT1 mRNA quantification by qRT-PCR analysis, samples from the Set A of patients were used (*Set A*). A second analysis was conducted with the Human Endogenous Control Plate (#4367563, Applied Biosystems) using the *Set B *of samples. A third analysis included the clinical validation of the selected optimally performing reference genes and was carried out on Sets A+C primary tumor samples and their paired non-malignant lung tissues.

Non-malignant human bronchial epithelial (NHBE) and small airways epithelial cells (SAEC), and seven NSCLC cell lines (NCI-H460, NCI-H1385, NCI-H157, NCI-H1648, NCI-H23, NCI-H441 and SK-MES-1) were used. Non-tumor cells were obtained from CAMBREX (NJ, USA), and tumor cells were obtained from ATCC (VA, USA). Non-malignant cells were grown in Bronchial Epithelial Cell Basal Medium (CAMBREX, NJ, USA), and NSCLC cell lines in RPMI-1640 supplemented with 10% fetal calf serum (Invitrogen, Carlsbad, CA).

### RNA extraction and qRT-PCR

Total RNA was isolated using the AllPrep DNA/RNA mini Kit (Qiagen, CA, USA) as described by the manufacturer. RNA concentrations and the A_260_/A_280 _ratio were measured with a NanoDrop^® ^ND-1000 (NanoDrop Technologies, Montchanin, DE, USA). The threshold inclusion values for the RNA samples were > 1.90 for the A_260_/A_280 _ratio. The absence of contaminating DNA was analyzed by running the samples through 2% agarose gels. RNA quality was also determined in a Bioanalyzer platform (Agilent, CA, USA). Two micrograms RNA were reverse transcribed. Before transcription, RNA was denatured for 5 min at 65°C followed by cooling on ice. First strand cDNA synthesis was carried out with SuperScript™ III Reverse Transcriptase (Invitrogen) and random primers (Invitrogen) in a total volume of 20 μl. Reverse transcription was performed at 42°C for 1 h followed by 72°C for 15 min. Finally, RNase H was added to the reaction mixture for 20 min at 37°C. cDNA was stored at -80°C until RT-PCR analysis. Each RNA sample was controlled for genomic DNA contamination by a reaction mix without reverse transcriptase addition. All cDNAs were diluted 1:10 before being used as PCR template.

Measurement of the expression of candidate genes was performed with the *TaqMan*^® ^*Low Density Human Endogenous Control Panel *(Applied Biosystems), according to the manufacturer's protocol. This plate contains sixteen human endogenous candidate genes (Table 1). qRT-PCR was performed with an Applied Biosystems 7900HT Fast Real-time PCR System. PCR efficiencies were calculated according to Rasmussen [15] and the standard curves generated in the qRT- PCR were plotted as Ct values versus logarithms of the given concentrations of the DNA templates.

**Table 1:** Genes included in the TaqMan^® ^low density human endogenous control panel

**Gene symbol**	**Gene name**	**Function**
18S	18S Ribosomal RNA	One 18S molecule makes the small subunit of the ribosome
ACTB	Beta-Actin	Cytoskeletal structural protein
B2M	Beta-2-Microglobulin	Beta-chain of major histocompatibility complex class I molecules
GAPDH	Glyceraldehyde-3-phosphate dehydrogenase	Oxidoreductase in glycolysis and gluconeogenesis
GUSB	Beta-Glucuronidase	Degradation of dermatan and keratan sulfates
HMBS	Hydroxymethylbilane synthase	Heme synthesis, porphyrin metabolism
HPRT1	Hypoxanthine phosphoribosyl transferase	Purine synthesis in salvage pathway
IPO8	Importin8	Function in nuclear protein import
PGK1	Phosphoglycerate kinase	Glycolytic enzyme
POLR2A	RNA Polymerase II	Catalyzes the RNA synthesis from DNA
PPIA	Peptidylprolyl isomerase A	Catalyzes the cis-trans isomerization of proline imidic peptide bonds in oligopeptides, accelerating folding
RPLP0	Ribosomal large P0	Ribosome biogenesis and assembly
TBP	TATA binding protein	General RNA polymerase II transcription factor
TFRC	Transferrin receptor	Cellular uptake of iron
UBC	Ubiquitin C	Protein degradation
YWHAZ	Tyrosinmonooxygenase/Tryp-tophanmonooxygenase activation protein	Signal transduction by binding to phosphorylated serine residues on a variety of signaling molecules

### Determination of gene stability

To evaluate suitability of candidates as reference genes, we applied two powerful previously published Microsoft Excel-based applications: 1) GeNorm [16], which calculates gene stability as the standard deviation (SD) of the log_2_-transformed expression ratios of each reference gene. The program is available on the Internet . Ct values were converted into relative quantities for analysis with GeNorm, considering the PCR efficiencies of the genes. 2) NormFinder [17], that uses a model-based approach to estimate expression stability based on intra- and intergroup variations for candidate endogenous control genes. It is also freely available on the Internet .

### Analysis of the absolute variation of Ct values

Ct variations were expressed as ΔCt, the difference between Ct Normal and Ct Tumor (ΔCt = Ct Normal-Ct Tumor). To analyze the absolute variation of Ct values, we calculated the mean and the standard error of the mean (SEM) of absolute values of ΔCt ("|ΔCt|") for each gene.

### Microarray data analysis

Three lung cancer patient microarrays (HuGene-FL, HG-U95A, HG-U133A), previously described [18-20], were analyzed. The raw datasets are publicly available at: . For analysis, significant differences in a specific reference gene candidate expression, between normal and tumor samples from patients (lung adenocarcinomas and squamous cell lung carcinomas) were identified by ANOVA. p-values < 0.05 were considered statistically significant.

### Statistical analysis

All statistical evaluations were carried out using the SPSS software package. Correlations between genes were determined by Pearson's test. All p-values < 0.01 were considered statistically significant in this analysis.

Normal distributions were assessed with the Shapiro-Wilk's W test. Differences in gene expressions between nonmalignant and malignant samples were calculated by the Student's t test for paired data with normal distribution, or by Wilcoxon's test for paired data following non-parametric distribution. p-values were considered significant when p < 0.05.

## Results

### HPRT1 and GAPDH, two genes commonly used for normalization, are inappropriate reference genes for human lung tissue analyses

**Figure 2:**
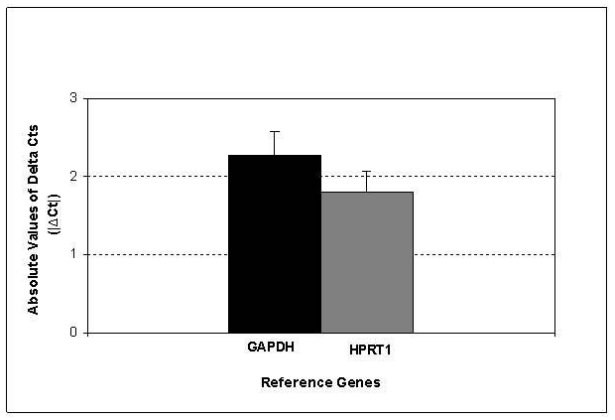
**Variations of GAPDH and HPRT1 expression levels**. Mean ± SEM of absolute values of ΔCt (|ΔCt| = |Ct Normal-Ct Tumor|) of the two commonly used reference genes (GAPDH and HPRT1) in paired lung clinical samples (Set A).

GAPDH and HPRT1 have been recommended as suitable reference genes for lung cancer research [21]. Thus, we first evaluated expression levels of these genes in the Set A samples. The analysis revealed that gene mRNA levels were significantly higher in tumors than in non-malignant tissues for both genes: GAPDH (p = 0.0001) and HPRT1 (p = 0.00003). Ct variations (ΔCt = Ct Normal-Ct Tumor) were calculated for each gene (Supplemental Table 2, Additional file 1). Ideally, a good reference gene should have |ΔCt| values close to zero with low SEM. However, as shown in Figure 2, neither GAPDH (|ΔCt| = 2.27 ± 0.31) nor HPRT1 (|ΔCt| = 1.81 ± 0.26) showed this pattern.

We also performed a statistical analysis of three lung cancer microarrays previously published [18-20]. The ANOVA analysis (p < 0.05) confirmed that there was a significant increase in both GAPDH and HPRT1 expression levels in tumor samples compared to normal tissues (Supplemental Table 3, Additional file 1). In summary, in lung clinical samples, as described in several other cancers [22-25], the expression of the two most commonly used reference genes is heterogeneous and, consequently, not valid for gene expression normalization.

### Variable expression of sixteen endogenous control genes in both lung cell lines and clinical samples

In the next experiments, we used the *Low Density Endogenous Control Panel *on human samples and cultured cells, with the goal of identifying suitable genes for normalization. We first analyzed the efficiency of the PCR assay. The linear correlation coefficient (R^2^) of the standard curves of all the genes ranged from 0.9942 to 0.999. Based on the slopes of the standard curves, the amplification efficiencies of the standards were from 91% to 100%, which were derived from the formula E = (10^1/-slope ^-1) × 100 [15]. The Ct values of the 16 genes in all the samples were within 10.7 and 35.3 cycles.

Differential expression levels and dispersion of individual Ct values from the mean Ct value were calculated for the 16 genes (Table 2). Except for 18S, which was the gene with the highest expression, all the other candidates showed Cts ranging from 19 to 33. In NSCLC cell lines, non-malignant cells, and lung tumors, the gene with the lowest mRNA levels was TBP, whereas the gene with the highest expression was ACTB. However, in normal clinical samples, HMBS showed the lowest level of expression (Ct = 32.68 ± 0.76), and B2M was highest expressed transcript (Ct = 22.94 ± 0.75) (Table 2).

**Table 2:** Comparison of mean cycle threshold (Ct ± SD) values across different sample groups

	**Cultured cells**		**Clinical samples**	
	**Tumor**	**Normal**	**Tumor**	**Normal**
**18S**	12.88 ± 0.61	11.24 ± 0.73	12.60 ± 1.62	13.79 ± 1.00
**ACTB**	22.20 ± 1.07	19.30 ± 0.26	23.07 ± 1.36	23.59 ± 1.15
**B2M**	24.99 ± 0.51	23.46 ± 0.43	23.21 ± 1.18	22.94 ± 0.75
**GAPDH**	22.61 ± 0.72	20.64 ± 0.00	24.48 ± 1.92	27.13 ± 1.11
**GUSB**	29.36 ± 0.73	28.61 ± 0.36	28.81 ± 1.36	29.77 ± 1.04
**HMBS**	29.23 ± 1.11	28.41 ± 0.45	30.89 ± 1.47	32.68 ± 0.76
**HPRT1**	27.07 ± 0.71	26.20 ± 0.40	29.10 ± 1.90	30,63 ± 0.97
**IPO8**	29.65 ± 1.19	28.97 ± 0.03	30.43 ± 1.37	30.91 ± 0.97
**PGK1**	24.97 ± 0.83	23.15 ± 0.32	25.23 ± 1.63	27.56 ± 0.92
**POLR2A**	27.95 ± 0.93	26.37 ± 0.06	28.55 ± 1.32	29.58 ± 1.06
**PPIA**	23.16 ± 0.82	21.49 ± 0.23	24.37 ± 1.47	26.00 ± 1.07
**RPLP0**	23.01 ± 0.71	21.02 ± 0.37	24.34 ± 1.82	26.01 ± 0.98
**TBP**	30.95 ± 1.75	29.00 ± 0.96	31.79 ± 2.12	32.59 ± 1.16
**TFRC**	26.71 ± 0.76	26.64 ± 0.13	27.94 ± 2.18	28.65 ± 1.06
**UBC**	23.11 ± 0.62	20.83 ± 0.10	23.82 ± 2.29	24.78 ± 1.48
**YWHAZ**	29.25 ± 0.68	26.72 ± 0.28	29.47 ± 2.05	31.06 ± 0.85

The SD of Ct values for each control gene was also calculated in the samples (Table 2). For NSCLC cell lines, B2M (SD = 0.51) expression showed the lowest SD. On the contrary, in non-malignant cells, GAPDH (SD = 0.00) was the gene with the lowest variability. B2M had the lowest SD for both tumor and normal clinical samples (1.18 and 0.75, respectively).

Because of the different patterns of expression of cultured cells and clinical samples, we decided to conduct further analyses separately. In the search for the most stable reference candidates, the gene expression stability was analyzed with *GeNorm *and *NormFinder *softwares.

### In lung cell lines, five genes (including GAPDH and HPRT1) perform optimally as endogenous control genes

As explained in *Materials and Methods*, *GeNorm *and *NormFinder *are two mathematical tools recently developed to identify expression stability of a set of candidate genes. The model-based approach (*NormFinder*) selects the candidates with minimal combined inter- and intra-group expression variation. The pairwise comparison approach (*GeNorm*) selects genes with a low intra-group variation and roughly the same no vanishing intergroup variation. *GeNorm *calculates the gene expression stability measure "M" of one gene, based on the average pairwise variation between all studied genes. The lowest M values characterize genes with the most stable expression.

Expression stabilities were first evaluated with *GeNorm *in cell lines (Table 3). In the ranking of expression stability, these genes were top-classified (*M *< 0.5): PPIA and RPLPO>GAPDH>18S>HPRT1. Genes with middle stability (0.5 <*M *< 0.7) were GUSB>POLR2A>HMBS>PGK1>UBC>ACTB. The less stable genes were B2M, TFRC, YWHAZ, IPO8 and TBP, whose *M *values were higher than 0.7 (Table 3).

**Table 3:** Candidate reference genes for normalization of qRT-PCR (in lung cultured cells) ranked according to their expression stability by GeNorm and NormFinder programs

**GeNorm**			**NormFinder**	
**Genes**	**Stability value**		**Genes**	**Stability value**
**PPIA**	0.377	Most stable genes	**PPIA**	0.078
**RPLPO**	0.377		**RPLP0**	0.199
**GAPDH**	0.387		**HPRT1**	0.232
**18S**	0.430		**GAPDH**	0.244
**HPRT1**	0.473		**18S**	0.257
GUSB	0.509		GUSB	0.328
POLR2A	0.547		POLR2A	0.407
HMBS	0.580		UBC	0.408
PGK1	0.614		PGK1	0.420
UBC	0.644		HMBS	0.425
ACTB	0.681		ACTB	0.508
B2M	0.719		B2M	0.529
TFRC	0.761		TFRC	0.583
YWHAZ	0.801		YWHAZ	0.631
IPO8	0.852		IPO8	0.758
TBP	0.917	Least stable genes	TBP	0.863

Using *NormFinder *with arbitrary cut-off values of 0.4 and 0.6 in the cell lines, the most stable genes were PPIA>RPLPO>HPRT1>GAPDH>18S>GUSB (Table 3). Genes with intermediate stability included POLR2A>UBC>PGK1>HBMS>ACTB>B2M>TFRC. Finally, YWHAZ, IPO8, and TBP were the least stable genes. Considering results from both softwares, PPIA, RPLPO, GAPDH, HPRT1, and 18S can be considered accurate reference genes in lung cancer cell lines.

We further determined correlations between genes by Pearson's test, considering statistically significant p-values < 0.01 (supplemental Text S1 and supplemental Table 4, Additional file 1). Interestingly, our five candidate genes (PPIA, RPLPO, GAPDH, HPRT1, and 18S) exhibited a very strong correlation among themselves (r = 0.949–0.814).

### Determination of a set of five candidate genes to be used as reference genes in clinical samples

In clinical samples, *GeNorm *identified the following genes with *M *values < 0.5 (very stable): IPO8, ACTB>POLR2A>18S; genes with *M *values ranging from 0.5 to 0.7 included PPIA>HMBS>RPLPO>YWHAZ. The group with *M *values >0.7 (not suitable for normalization) were PGK1>HPRT1>TBP>GAPDH>UBC>B2M>GUSB>TFRC (Table 4). Using *NormFinder *in clinical samples we observed that the most stable candidates were the following: PPIA>POLR2A>18S>HMBS>RPLPO>IPO8>ACTB>YWHAZ, with stability values < 0.4 (Table 4). Genes with stability values between 0.4 and 0.6 were HPRT1, TBP, PGK1, UBC, GUSB, and TFRC. The worse stable genes were GAPDH and B2M.

**Table 4:** Rank of expression stability (in lung tissues) calculated by GeNorm and NormFinder softwares

**GeNorm**			**NormFinder**	
**Genes**	**Stability value**		**Genes**	**Stability value**
**IPO8**	0.245	Most stable genes	PPIA	0.253
**ACTB**	0.245		**POLR2A**	0.267
**POLR2A**	0.344		**18S**	0.276
**18S**	0.466		HMBS	0.318
PPIA	0.530		RPLP0	0.323
HMBS	0.564		**IPO8**	0.336
RPLP0	0.599		**ACTB**	0.360
YWHAZ	0.658		YWHAZ	0.383
PGK1	0.717		HPRT1	0.430
HPRT1	0.774		TBP	0.474
TBP	0.823		PGK1	0.484
GAPDH	0.872		UBC	0.510
UBC	0.931		GUSB	0.535
B2M	0.984		TFRC	0.586
GUSB	1.045		GAPDH	0.653
TFRC	1.101	Least stable genes	B2M	0.701

Consequently, the best three genes using *GeNorm *were IPO8, ACTB, and POLR2A, whereas the best three candidates using *NormFinder *were PPIA, POLR2A, and 18S. We conclude from both analyses that IPO8, ACTB, POLR2A, 18S and PPIA are suitable reference genes for lung biopsies. Furthermore, these genes exhibited a high *Pearson *correlation among themselves (r = 0.981–0.857) (supplemental Text S2 and Supplemental Table 6, Additional file 1). Moreover, IPO8 was the gene with the highest number of significant correlations (supplemental Table 7, Additional file 1). Since we had two different top-ranked candidates, depending on the type of analysis, we carried out further experiments to identify the best normalizing gene among the best five genes.

**Figure 3:**
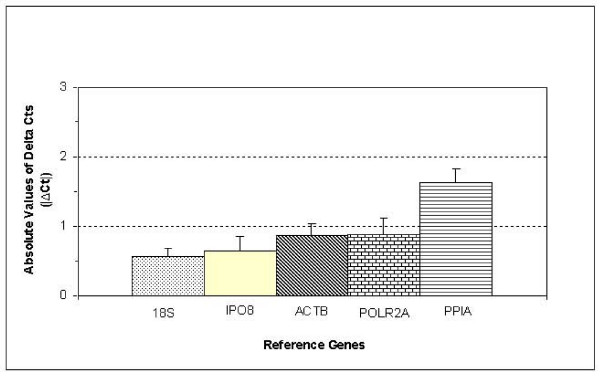
**Average of absolute values of ΔCt of IPO8, ACTB, POLR2A, 18S and PPIA**. Mean ± SEM of |ΔCt| (=|Ct Normal-Ct Tumor|) of five selected optimally performing reference genes (IPO8, ACTB, POLR2A, 18S and PPIA) in paired lung clinical samples (Set C).

This analysis was performed using the Set C sample, and calculating the means ± SEM of |ΔCt| (|Ct Normal-Ct Tumor|) for the aforementioned five genes (Figure 3). The ranking of these genes according to this criterion was as follows: 18S(0.563 ± 0.123)>IPO8(0.638 ± 0.222)>ACTB(0.875 ± 0.153)>POLR2A(0.878 ± 0.242)>PPIA(1.633 ± 0.201). The tests of paired data showed no significant differences between normal and malignant tissues for IPO8 (p = 0.877), whereas significant differences were found for all the other genes: PPIA (p = 0.000), 18S (p = 0.007), POLR2A (p = 0.011) and ACTB (p = 0.046). Therefore, IPO8 can be considered the best candidate, taking into account all these criteria (high stability, low mean ± SEM of |ΔCt| with no significant differences between paired samples, and number of correlations).

### IPO8 as the most accurate reference gene for clinicopathological specimens

In order to further validate IPO8 as the best control gene for lung tissues, a third analysis was performed in Sets A+C samples. In addition, we used this analysis to reexamine PPIA as a putative control gene (since it was top-classified by *NormFinder*) and to further validate the inaccuracy of GAPDH and HPRT1 as normalizing genes for clinicopathological lung specimens. In this case, we used |ΔCt| ± SEM, but not expression stability based on *GeNorm *and *NormFinder*, because the validity of this latter analysis relies on examining a large number of genes (typically 5 to 10) [16].

**Figure 4:**
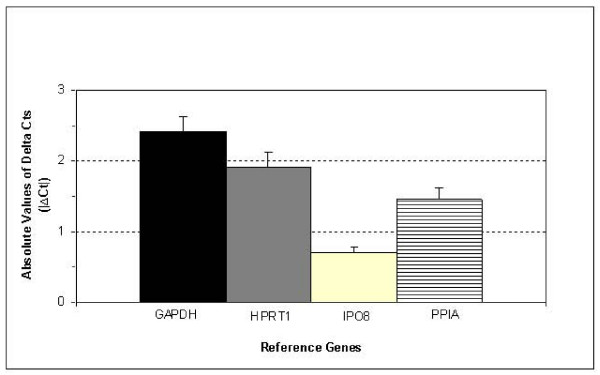
**IPO8 as the most accurate reference gene in lung specimens**. Average (mean ± SEM) of |ΔCt| of the two commonly used reference genes (GAPDH and HPRT1), PPIA and the novel reference gene IPO8 in paired lung clinical samples (Sets A+C).

The test of paired data revealed no significant differences between normal and malignant samples for IPO8 mRNA levels (|ΔCt| = 0.70 ± 0.09). On the contrary, the three other genes showed significantly different |ΔCt| values when comparing non-malignant with malignant tissues (Figure 4). IPO8 was also the gene with the lowest SEM (Figure 4). Therefore, these results confirmed that IPO8 is the best reference gene for normalizing lung tissue samples. PPIA showed |ΔCt| = 1.45 ± 0.17, and GAPDH and HPRT1 had the highest difference in |ΔCt| (on average) between normal and tumor samples: 2.42 ± 0.20 for GAPDH and 1.91 ± 0.21 for HPRT1.

## Discussion

The identification of novel diagnostic tools and therapeutic targets for lung cancer relies on the accurate normalization with reference genes whose expression remains constant in both normal and malignant tissues. Stringent requirements for selecting endogenous controls are essential, and the task of identifying normalization genes is not trivial. Several recent papers have demonstrated that classical "housekeeping" genes such as GAPDH, HPRT1, and ACTB (β-actin) are inaccurate to normalize different types of clinical samples [23,25-27] In the present study, we have analyzed the expression of a panel of 16 genes in lung cancer cell lines and biopsies, with the goal of identifying the most accurate candidate to be used as a reference gene. The main finding of our study is the identification of importin-8 (IPO8) as a very robust reference gene for lung clinical specimens, which could become the gold-standard endogenous gene for lung tissues.

One first conclusion is that GAPDH and HPRT1, the two most commonly reference genes used in the literature, are not suitable for the normalization of gene expression lung biopsies. Nonetheless, our data also show that the use of both genes is perfectly appropriate for expression studies using lung cell lines. In contrast, in clinical specimens we have clearly shown a significant increase in GAPDH and HPRT1 mRNA levels in tumors (as compared to normal matched tissues) and low expression stability. Despite GAPDH was widely used in the past, its use as a reference gene has recently been challenged in the majority of tumor types, including melanoma [22], liver [23], bladder [24], renal cancer [25], prostate [26], gastroesophagic and pancreatic cancer [27], and colon adenocarcinoma [28].

*In vitro *assays have demonstrated that GAPDH contributes to diverse cellular functions related to glycolysis, nuclear RNA export, DNA replication and repair, exocytosis, and cytoskeletal organization [29]. GAPDH was also suggested to play a role in the pathogenesis of cancer [29]. Remarkably, antisense oligodeoxynucleotides targeting GAPDH inhibit cell proliferation and induce apoptosis in cervical carcinoma cells. Taken together our results and data from the literature, in spite of a previous study proposing GAPDH as a good normalizing gene for lung biopsies [21], we strongly suggest not to use this gene for gene expression normalizing purposes in lung.

In our search for the most accurate gene to normalize lung specimens, we ranked the 16 candidate genes according to expression stability, and mean of |ΔCt| ± SEM values. According to *GeNorm*, the best three genes in terms of expression stability were IPO8, ACTB, and POLR2A, whereas using *NormFinder *PPIA, POLR2A, and 18S were top-classified. From both analyses, we consequently proposed a set of five genes (IPO8, ACTB, POLR2A, 18S and PPIA) as suitable reference genes for lung specimens. Considering the lowest |ΔCt|, 18S was top-ranked (0.563 ± 0.123), closely followed by IPO8 (0.638 ± 0.222). However, statistical comparison of expression levels between normal and malignant tissues found no differences exclusively for IPO8, but not for any other gene. In addition, expression of IPO8 strongly correlated (r^2 ^> 0.9) with that of 18S, ACTB, and POLR2A. IPO8 had also the highest number of gene correlations. In view of all these results we conclude that IPO8 is the most robust reference gene for lung cancer studies. Indeed, we further validated the accuracy of IPO8 as a reference gene in a different set of samples and found again that Ct values for normal samples were statistically similar to those of tumors.

Interestingly, IPO8 has never been proposed as a potential reference gene in cancer research. Importin 8 (IPO8), a gene located at 12p11.21, which encodes a protein of 1037 aminoacids, is a member of a class of approximately 20 potential Ran targets that share a sequence motif related to the Ran-binding site of importin-beta. This protein binds to the nuclear pore complex and, along with RanGTP and RANBP1, inhibits the GAP stimulation of the Ran GTPase. The importin-alpha/beta complex and the GTPase Ran mediate nuclear import of proteins with a classical nuclear localization signal [30].

Despite its accuracy in the normalization of lung clinical samples, IPO8 is not the best option for *in vitro *studies. Although |ΔCt| for IPO8 was also very low in cell lines, its expression is not stable (according to *Genorm *and *NormFinder *analysis). In cell lines, PPIA was top-classified in terms of expression stability, followed by RPLPO, 18S, and HPRT1. 18S was included in the group of optimally performing endogenous genes in all the analyses of our study, for both cell lines and clinical samples. Therefore, 18S could be an alternative to IPO8 when a study required the use of cell lines and biopsies with a single reference gene. However, 18S rRNA levels are extremely high (Ct values between 12.6–12.8) in comparison to other target housekeeping mRNAs, which may increase the risk of introducing quantification errors. In addition, some studies have suggested that mRNA transcripts should not be normalized with a ribosomal RNA, because of their unrelated expression mechanisms [31].

Systematic comparisons of gene sets in different types of tumors have recently led to the selection of a variety of optimal reference genes: SDHA and TBP for bladder [24], 18S for gastric and colorectal [27], PPIA and TBP for renal [25], HPRT1 for prostate [26], SFRS4 for hepatocellular carcinoma [23], or B2M for colon adenocarcinoma [28]. It seems clear that a single definitive universal reference gene has not been identified yet, and may be very difficult to find, as tissue specific gene expression is the basis for tissue and organ differentiation. Consequently, appropriate control genes for each specific tumor type need to be selected among a variety of candidates, using stringent mathematical criteria.

## Conclusion

We can draw several important conclusions from our study: a) GAPDH and HPRT1 are not suitable genes to normalize lung specimens but are appropriate when using lung cell lines; b) The best performing reference genes for lung cell lines are not coincident with those of clinical samples; c) PPIA is a novel reference gene for lung cell lines; d) Finally, and most importantly, we have described for the first time that Importin-8 is the best performing gene to normalize clinicopathological lung samples and should be considered as the main option when using lung biopsies. We believe that this finding will help further studies to normalize potential new targets for diagnosis and treatment of lung cancer.

## Abbreviations

cDNA: complementary DNA; NSCLC: non-small cell lung cancer; RT-PCR: reverse transcription-PCR.

## Authors' contributions

PAN and JA were the main contributors to the manuscript, carrying out most of the experiments, data analysis, design, interpretation of the results, and writing most of the manuscript. MDL and JGR collected the samples and made critical contributions to the analysis of results. MJR and BAS participated in the study design and performed part of the experiments. DB, IV, MJP, and RP contributed to the data analysis. LMM and AC participated in the study design, supervised the work and partially wrote the manuscript. All authors read and approved the manuscript.

## Supplementary Material

Additional file 1 **Supplementary material** Click here for file
