# Supplementary material for: Identification of Importin 8 (IPO8) as the most accurate reference gene for the clinicopathological analysis of lung specimens
Source: BMC Mol Biol. 2008 Nov 17;9:103. doi: 10.1186/1471-2199-9-103 (PMC2612021; doi:10.1186/1471-2199-9-103)
Supplement: Additional file 1 — Supplementary material [file 1471-2199-9-103-S1.doc]

**Identification of Importin 8 (IPO8) as the most accurate housekeeping gene for the clinicopathological analysis of lung specimens**

Paul A. Nguewa^1^, Jackeline Agorreta^1^, David Blanco^1^, Maria Dolores Lozano^2^, Javier Gomez-Roman^3^, Blas A. Sanchez^4^, Iñaki Valles^1^, Maria J. Pajares^1^, Ruben Pio^1^, Maria Jose Rodriguez^4^, Luis M. Montuenga^1#*^, Alfonso Calvo^1#^

^1^ Division of Oncology, Center for Applied Medical Research (CIMA), University of Navarra. Avda. Pio XII, 55. 31008 Pamplona, Spain.

^2^ Department of Pathology. University Hospital of Navarra. Avda. Pio XII, 36. 31008 Pamplona, Spain

^3^ Department of Anatomical Pathology, Marqués de Valdecilla University Hospital, Medical Faculty, University of Cantabria, Santander, Spain.

^4^ Research Department. Ingenasa

#: Contributed equally to this paper

Email addresses:

PAN: panguewa@unav.es

JA: jagorreta@unav.es

DB: dblanco@unav.es

MDL: mdlozano@unav.es

JG-R: apagrj@humv.es

BAS: bsanchez@ingenasa.es

IV: ivalles@unav.es

MJP: mpajares@unav.es

RP: rpio@unav.es

MJR: mjrodriguez@ingenasa.es

LM*: lmontuenga@unav.es

AC: acalvo@unav.es

*** Corresponding author**:

**“Online Data Supplement”**

**Sources of support**:

This work has been funded by “UTE project CIMA”, ISCIII-RETIC RD06/0020 grant; Cenit project ONCNOSIS, Government of Navarra. P.A.N. was supported by a Spanish Torres-Quevedo fellowship (PTQ05-01-01084) and J.A. by a fellowship from the “Instituto de Salud Carlos III”, Ministry of Health, Spain.

**Supplemental Table 1:** Clinical characteristics of patients with lung cancer

**Supplemental Table 2** Absolute values of ∆Ct (|∆Ct|=|Ct Normal-Ct Tumor|) of GAPDH and HPRT1 in paired lung clinical samples (Set A). Mean and Standard error of mean (SEM) were calculated.

**Supplemental Table 3** ANOVA test of three lung cancer patient microarrays (HuGene-FL, HG-U95A, HG-U133A). The raw datasets are publicly available at the website: [www.affymetrix.com/products/arrays/index.affx](http://www.affymetrix.com/products/arrays/index.affx). (*) p-values <0.05 were considered statistically significant.

**Supplemental Text 1*. Correlation between endogenous control genes in lung tumor cell lines***

The four strongest correlations (**p<0.01) (See supplemental Table 4, in bold) were found between RPLPO and ACTB (r=0.970), PPIA and ACTB (r=0.969), PPIA and HPRT (r=0.949), and GAPDH and POLR2A (r=0.944). In addition, we also evaluated the number of significant correlations (**p<0.01) of each gene among the sixteen others, in lung cell lines (See supplemental Table 5). We then classified the genes in three groups depending on the number of significant correlation (NC). The ones with very good and good numbers of significant correlation were: PPIA and ACTB (NC=11/16); HPRT1, GAPDH, RPLPO (NC=9/16); and 18S (NC=8/16). Those with bad significant correlation were: UBC (NC=7/16); PGK1, POLR2A (NC=6/16), GUSB, TBP (NC=5/16), and HMBS (NC=4/16). The last group included genes with the lowest NC, thus indicating the worse correlations with other reference genes: YWHAZ (NC=3/16); B2M (NC=2/16); IPO8 and TFRC (NC=1/16).

**Supplemental** **Table 4** Correlation between sixteen reference genes in lung cancer cell lines.

Data are represented as coefficients (r-value) between each two housekeeping genes. HKG: housekeeping genes. (**) p-Values are considered significant when p<0.01.

**Supplemental Table 5** Number of significant correlations (NC) (**p<0.01) of each gene over all the sixteen endogenous control genes in lung cancer cell lines.

**Supplemental Text S2*. Correlation between endogenous control genes in normal and tumoral lung tissues***

In Supplemental Table 6, correlations between endogenous control genes in lung tissues are represented as coefficients (r-value). Most of endogenous control genes were strongly correlated. The strongest correlations (**p<0.01) (Table S5, in bold) were between IPO8 and ACTB (r=0.981), PPIA and RPLPO (r=0.969), POLR2A and 18S (r=0.962), IPO8 and POLR2A, PPIA and HMBS, GAPDH and PGK1 (r=0.957), HPRT and GAPDH (r=0.956), PPIA and POLR2A (r=0.954), and PPIA and PGK1 (r=0.953). The evaluation of the numbers of significant correlation (**p<0.01) of each gene among the sixteen others in lung tissues (See Supplemental Table 7) revealed two groups of genes. Except for three genes, all the others exhibited very good or good NC, ranging from 14/16, to 11/16. The best one was IPO8 (NC=14/16). PPIA, ACTB, HMBS, POLR2A, 18S, and RPLPO had NC=13/16; HPRT, PGK1, TBP, and YWHAZ: NC=12/16; and finally, GAPDH and UBC, NC=11/16. However, the group with the worse numbers of significant correlation was: B2M and TFRC (NC=3/16), and GUSB (NC=1/16).

Consequently, this analysis shows that IPO8, 18S, ACTB, POLR2A, PPIA, HMBS, and RPLPO exhibit a strong correlation expression among them (r=0.981-0.953) as well as high numbers of significant correlations (NC=14-13 over the sixteen genes).

**Supplemental** **Table 6** Correlation between sixteen reference genes in lung cancer tissues.

Data are represented as coefficients (r-value) between each two housekeeping genes. HKG: housekeeping genes. (**) p-Values are considered significant when p<0.01.

**Supplemental Table 7** Number of significant correlations (NC) (**p<0.01) of each gene over all the sixteen endogenous control genes in lung cancer tissues.
